# A Confidence‐Based Multibody Kinematics Optimization for Markerless Motion Capture: A Proof of Concept

**DOI:** 10.1002/cnm.70079

**Published:** 2025-08-12

**Authors:** Anaïs Chaumeil, Pierre Puchaud, Antoine Muller, Raphaël Dumas, Thomas Robert

**Affiliations:** ^1^ Univ Eiffel, Univ Lyon 1, LBMC UMR T_9406 Lyon France; ^2^ Laboratoire de Simulation et Modélisation du Mouvement, Département de Kinésiologie Université de Montréal Laval Canada

## Abstract

Multi‐camera markerless motion capture commonly triangulates 3D points from 2D keypoint positions in multiple camera views, then applies a multibody kinematics optimization (MKO) to incorporate biomechanical constraints. However, standard pipelines neglect the 2D confidence heatmaps generated by human pose estimation networks. We hypothesized that performing MKO in 2D camera planes would make it more robust to missing keypoints and allow us to obtain better accuracy. 2D confidence heatmaps were used to maximize available information. To test this, we first model each network‐derived heatmap as a 2D Gaussian function characterized by its center, amplitude, and standard deviation. Second, we maximize the sum of these modeled confidences after projecting the biomechanical model into the camera planes. To demonstrate feasibility, we evaluated our method on data from two participants performing sit‐to‐stand, walking, and manual material handling, captured by a two‐camera setup, and simultaneously collected marker‐based data. Our Gaussian modeling of the heatmaps demonstrated a mean absolute difference of 0.011 compared to the original discrete maps, confirming its validity. In terms of 3D joint positions and angles, the confidence‐based MKO produced results similar to classical distance‐based methods. Notably, the confidence‐based approach overcame occultations: 89.3% of frames could only be obtained with the distance‐based MKO due to missing keypoints, while the confidence‐based MKO computed 100% of frames. These findings underscore the potential of using full 2D confidence heatmaps in markerless motion capture, especially under challenging conditions such as sparse camera setups.

## Graphical abstract

Using 2D confidence information in multibody kinematics optimization improved the robustness of markerless motion capture using two video cameras. The proposed method performs the optimization in 2D camera planes instead of in three dimensions, allowing for the computation of 3D kinematics even when 3D point reconstruction is impossible.

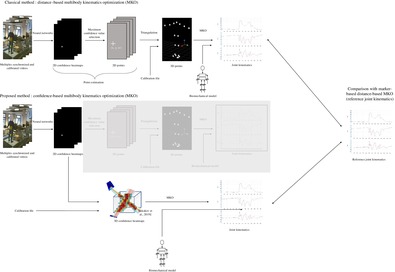

## 1 Introduction

In recent years, numerous markerless motion capture methods based on 2D human pose estimation (HPE) networks have been proposed in the biomechanics community [1, 2, 3, 4]. These methods come from a strong interest in this community to reduce the instrumentation of the participants when performing motion analysis. Contrary to marker‐based motion capture, which requires a large number of cameras to overcome occlusions [5], multi‐camera markerless motion capture associated with 2D pose estimation can be used with a significantly reduced number of cameras viewing the participant. Several studies have used only two cameras [4, 6, 7, 8], which is the minimum to be able to perform triangulation, also called Direct Linear Transform (DLT). Having only two cameras reduces the experimental burden associated with having multiple cameras, especially in ecological situations such as sports fields or work environments. The DLT uses camera calibration parameters and 2D keypoint positions to reconstruct the 3D point position [9]. However, using only two cameras to triangulate 2D keypoints comes with its challenges: the available information is limited, and if a 2D keypoint is not estimated, then its associated 3D position cannot be computed.

Multibody kinematics optimization (MKO) is used in biomechanics to obtain 3D kinematics based on experimental measurements. MKO allows defining the pose of segments while enforcing biomechanical constraints [10], which results in compensation for soft tissue artifacts in kinematic and dynamic computations [11]. Classically, the MKO minimizes the distance between experimentally obtained and model‐based 3D points. Thus, these methods based on 3D point reconstruction are sensitive to the non‐detection of 2D keypoints, in particular when using only two cameras [12]. An alternative could be to modify the MKO formulation to work on the 2D information available. We hypothesize that such a formulation would be more robust, as it would use information available in each camera's plane, even if the 3D reconstruction is impossible (e.g., if the keypoint is not detected on one of the two cameras).

When estimating 2D keypoints positions, most HPE networks generate 2D confidence heatmaps. Each pixel in these heatmaps is assigned a confidence score indicating the likelihood that the keypoint is located at that specific pixel. The keypoint's position is then derived by selecting the pixel with the highest confidence score. It has been proved that the confidence value associated with the keypoint's position can be of interest for 3D point reconstruction, by weighting the DLT [13]. However, this confidence value is local information, especially compared to 2D confidence heatmaps, which provide confidence information for the whole image. In particular, by using this local information, the distance between the experimentally obtained and model‐based points is given by an arbitrary metric (usually the $\ell^{2}$‐norm of the vector between these points), similar for every keypoints. Interestingly, this arbitrary metric could be replaced by the continuous 2D confidence heatmaps provided by the HPE. We thus made the hypothesis that using the whole confidence information contained in 2D confidence heatmaps could be beneficial to computing joint kinematics for markerless motion capture, for both improving robustness to missing keypoints and accuracy.

However, current MKO formulations are not designed to work on 2D heatmaps. In particular, heatmaps from neural networks are discrete. For the MKO to remain fast and efficient, the heatmaps need to be modeled as continuous functions so they can be differentiated and guarantee good convergence properties. This modified MKO will be referred to as confidence‐based MKO, distinguishing it from the conventionally used distance‐based MKO.

Therefore, the goal of this study is to present a proof‐of‐concept of a confidence‐based MKO method that leverages full 2D confidence heatmaps for markerless motion capture with two cameras. Evaluations will be carried out to evaluate its feasibility and provide insights into accuracy levels and eventual advantages to using this method. The study is organized in three sections (i) introduction of the new confidence‐based MKO method that leverages full 2D confidence heatmaps for markerless motion capture, (ii) validation of the modeling of these confidence heatmaps as continuous and differentiable 2D Gaussian functions, and (iii) preliminary assessment of the accuracy of the resulting 3D kinematics by comparing them against a trusted reference dataset from marker‐based motion capture. Our central hypotheses are that performing MKO in 2D camera planes and using the continuous confidence information from the heatmap improves robustness to missing information and that it would lead to a better accuracy. It should enable the method to recover 3D kinematics even when one 2D keypoint could not be estimated, which often happens in two‐camera setups.

## 2 Materials and Methods

In this section, we will first present the distance‐based and confidence‐based MKOs, and then we will develop the evaluations of these methods.

### 2.1 Multibody Kinematics Optimizations

This section presents the formalism selected for the distance‐based and confidence‐based MKOs, namely natural coordinates. The objective function associated with each MKO is then defined. The distance‐based MKO can be used with 3D points either obtained from video cameras or opto‐electronic systems.

#### 2.1.1 Natural Coordinates

Natural coordinates [14] need two points ($r_{\mathrm{Pi}}$ and $r_{\mathrm{Di}}$) and two unit vectors ($u_{i}$ and $w_{i}$) to parameterize each segment i. It results in a vector $Q_{i}$ of 12 natural coordinates, complemented by a set of six equations representing the rigid body constraints. Given a set of natural coordinates $Q_{i}$, the 3D position $X_{\mathrm{mod},j}$ of any point j of the segment i could be obtained using its interpolation matrix $N_{j,i}$:

(1) $$X_{\mathrm{mod},j}\left(Q\right)=N_{j,i}Q_{i}$$

where $Q={\left(Q_{1},Q_{2},\ldots ,Q_{n}\right)}^{T}$ is the natural coordinates of the whole model.

#### 2.1.2 Distance‐Based MKO

The idea of the distance‐based MKO is to match as closely as possible each experimental 3D position $X_{\exp,j}$ with its model‐derived 3D position $X_{\mathrm{mod},j}\left(Q\right)$ through the following minimization problem, solved for each frame with the optimization problem presented in Equation (2):

(2) $$\min_{Q}\sum_{j=1}^{m}\|X_{\exp,j}-X_{\mathrm{mod},j}\left(Q\right)\|2$$

$$s.t.h\left(Q\right)=0$$

with m the number of virtual points of the biomechanical model used to drive the model. $X_{\exp,j}$ can be obtained either by video cameras or opto‐electronic systems. The holonomic constraints $h\left(Q\right)$ rigidify and articulate the bodies together, and are also termed as kinematic constraints [15]. Additional inequality constraints forced the segment coordinate systems to remain direct by satisfying $\det\left(\left[u_{i},r_{P_{i}}-r_{D_{i}},w_{i}\right]\right)\geq 1$. The optimization is solved for each frame successively. For the first frame, the natural coordinates Q used to initiate the optimization are estimated from the experimental positions $X_{\exp}$. This estimation is expected to be a good initial guess but it does not satisfy the holonomic constraints. For the following frames, the natural coordinates obtained at the previous frame are used as an initial guess for the optimization of the current frame.

#### 2.1.3 Confidence‐Based MKO

The difference between the distance‐based and the confidence‐based MKOs lies in the objective function. Instead of matching the location of model‐derived points, it aims at maximizing (i.e., minimizing the inverse of) the sum of the confidence of each model‐derived point of the biomechanical model projected in each camera plane:

(3) $$\min_{Q}{\left(\sum_{j=1}^{m}\sum_{k=1}^{n}C(p(X_{\mathrm{mod},j}\left(Q\right),T_{k}))\right)}^{-1}$$

$$s.t.h\left(Q\right)=0$$

with $X_{\mathrm{mod},j}\left(Q\right)$ the 3D position of model‐derived point *j* and $T_{k}$ the intrinsic and extrinsic camera calibration parameters of camera *k*. The function *p* calculates the 2D position x of a point in the camera reference frame from its 3D position and the camera calibration parameters. *C* is a function computing the confidence value of a 2D point.

An estimate $\tilde{C}$ of the confidence value was calculated with a 2D Gaussian analytical, differentiable, and continuous function:

(4) $$\tilde{C}\left(x_{j,k}\right)={\tilde{A}}_{k}\exp\left\{-\left[\left(\frac{{\left\|x_{j,k}-{\tilde{x}}_{k}\right\|}^{2}}{2{{\tilde{\sigma }}_{k}}^{2}}\right)\right]\right\}$$

This modeling is common in the literature [16, 17], especially for the training of pose estimation networks. The function depended on four estimated parameters: the coordinates of the center of the 2D Gaussian ${\tilde{x}}_{k}$, the confidence value at the center of the Gaussian ${\tilde{A}}_{k}$ and a standard deviation ${\tilde{\sigma }}_{k}$ controlling the spread of the confidence area. Only one standard deviation was computed, as there was no reason for any direction to prevail over the others.

For each 2D heatmap, the center of the Gaussian ${\tilde{x}}_{k}$ was located at the pixel with the maximum confidence value. This maximum confidence value was then assigned as the confidence value at the Gaussian's center ${\tilde{A}}_{k}$. The standard deviation ${\tilde{\sigma }}_{k}$ of the Gaussian was estimated as the average of the standard deviations computed along *x* and *y* lines passing through the center. Specifically, the *x* standard deviation was the standard deviation of the confidence values in a 60 × 60‐pixel window along the *x* line passing through the center. Similarly, the *y* standard deviation was the standard deviation of the confidence values in the same window along the *y* line passing through the center. By its simplicity, this method to estimate the standard deviations of the Gaussian has a near‐zero computational cost.

### 2.2 Evaluation

#### 2.2.1 Experimental Data

Two participants were recruited for this study: a man (24 years old, 1.74 m, 58 kg) and a woman (29 years old, 1.57 m, 60 kg), both presenting no known musculoskeletal pathologies. Participants wore underwear and were equipped with a full‐body marker set consisting of 48 reflective markers placed over anatomical landmarks [18]. The protocol was reviewed and approved by the ethics review board of University Gustave Eiffel. Written informed consent was obtained from both participants.

**FIGURE 1:**
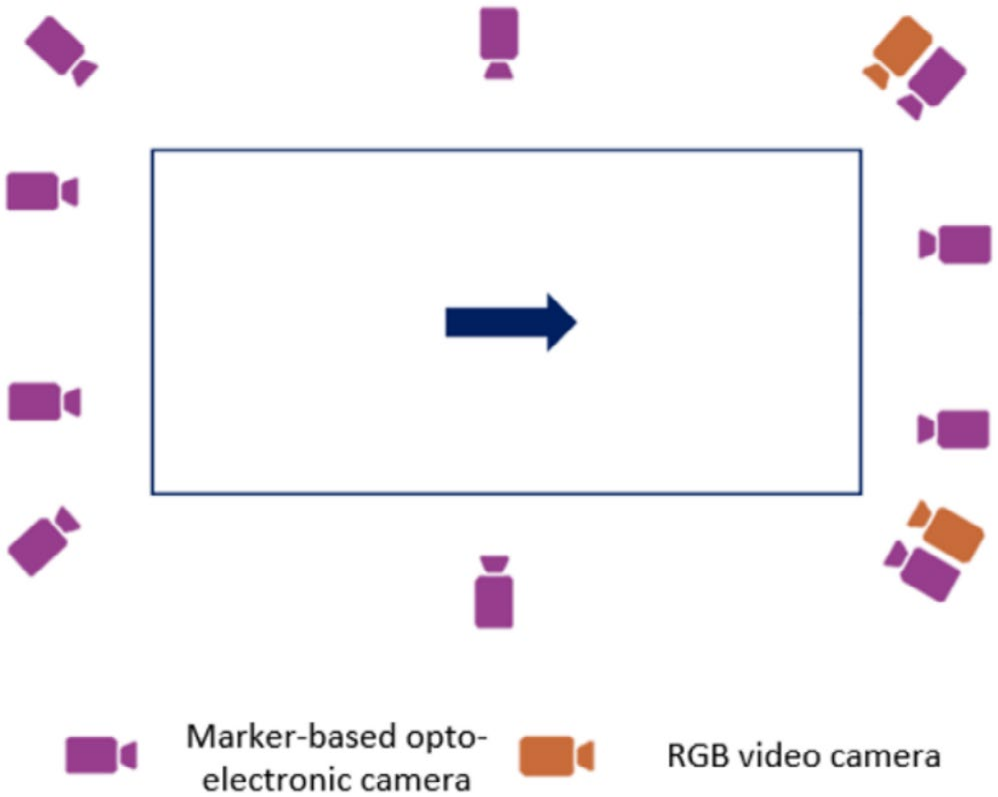
Position and orientation of the marker‐based opto‐electronic and RGB video cameras. The arrow represents the direction of movement.

**FIGURE 2:**
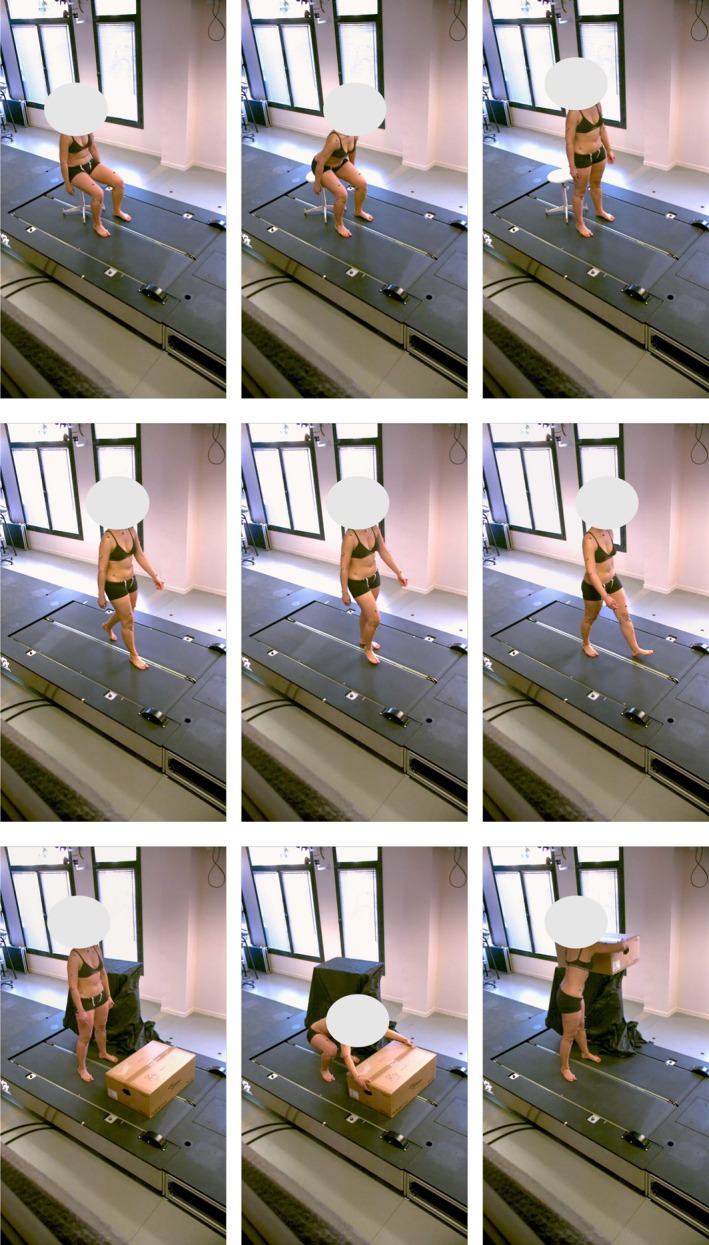
Illustration of the movements performed by the participants. Top row: Sit‐to‐stand. Middle row: Walking at self‐selected speed. Bottom row: Manual material handling task.

Data were collected using two complementary systems, synchronized through Qualisys Track Manager (QTM—Qualisys AB, Sweden). The marker‐based optoelectronic system comprised 10 Qualisys Miqus M3 cameras (120 Hz) distributed around the capture volume. The markerless system consisted of two Qualisys Miqus Video cameras (60 Hz, resolution 1920 × 1088 pixels) positioned between the frontal and sagittal planes (Figure 1). Participants performed three distinct tasks (Figure 2): a sit‐to‐stand motion, 10 s of walking at a self‐selected speed, and a manual material handling task that required transferring an empty cardboard box from the ground to a platform and then placing it back on the ground. Both marker and video data were made publicly available [19].

#### 2.2.2 Data Processing

Video data were processed with OpenPose (v1.7.0), using the *body_25b* model, net resolution of 368 × 656 and default scale number and render threshold parameters. 2D keypoints for the neck, shoulders, elbows, wrists, hips, knees, ankles, heels, big toes, and small toes were extracted. The resulting 2D confidence heatmaps for each keypoint were then modeled as continuous 2D Gaussian functions, according to the procedure described in Section 2.1.3. To evaluate the fidelity of this modeling step, the absolute differences between the estimated (i.e., OpenPose‐generated) and modeled heatmaps were computed. Comparisons were performed within a 60 × 60‐pixel window centered at the highest‐confidence pixel of each heatmap given by OpenPose. Two distributions of confidence differences were considered: one including all confidence differences for the 60 × 60‐pixel windows for each participant, task, keypoint and frame $\left(D_{60\times 60}\right)$, and one including only the maximum confidence difference of the 60 × 60‐pixel windows for each participant, task, keypoint and frame $\left(D_{\max}\right)$. Descriptive statistics (mean, maximum, 25th, 50th, 75th, and 99th percentile) were computed across both distributions $D_{60\times 60}$ and $D_{\max}$.

Three MKOs were employed to compute 3D kinematics. First, the proposed confidence‐based MKO utilized the parameters of the modeled 2D Gaussian functions (center position, maximum amplitude, and standard deviation) to incorporate the spatial distribution of confidence information into the optimization. Second, a distance‐based MKO was conducted on markerless data by reconstructing 3D points with a weighted DLT (wDLT [13]). wDLT could only be applied when keypoints from both cameras were estimated with a confidence value higher than the default threshold of 0.05 applied by OpenPose. Any gaps in keypoint trajectories, corresponding to null confidence, were filled by linear interpolation before triangulation if they were shorter than 10 consecutive frames. Finally, a distance‐based MKO was applied to marker data, serving as a reference. We implemented all three MKOs using BioNC [20], which utilizes CasADi [21] with its SX type, and an exact Hessian approximation. The optimization employed the IPOPT [22] interior point solver, using MUMPS as the linear solver. Tolerances were left at default values.

All MKOs relied on a human body model composed of a torso, pelvis, arms, upper arms, thighs, shanks, and feet. Hips and shoulders were represented as spherical joints, while elbows and knees were implemented as hinge joints. A six‐degree‐of‐freedom (DOF) joint linked the torso and pelvis. The model was built through a static pose. Joints in the markerless MKOs were defined directly from the estimated keypoints, whereas for the reference marker‐based MKO, joints were derived from marker positions [23].

From each MKO, the 3D positions of the shoulders, elbows, wrists, hips, knees, ankles, and mid‐toes were extracted, along with the hip, knee, ankle, shoulder, and elbow joint angles. Shoulder angles were expressed as azimuth and elevation, with 0° azimuth and 0° elevation corresponding to a fully extended arm along the torso. Hip angles were computed with the ZXY Euler sequence. To assess accuracy, both the confidence‐based and the distance‐based markerless MKOs were compared against the reference marker‐based MKO through 3D Euclidean distance errors of selected joint locations and joint angle errors at the hip, knee, ankle, shoulder, and elbow.

## 3 Results

### 3.1 Validation of the Gaussian Modeling

The difference between the estimated and modeled heatmaps was computed on an area of 60 × 60 pixels around the pixel with the maximum confidence, for the 2 participants, all keypoints, and each frame of the 3 movements performed. The mean difference of the $D_{60\times 60}$ distribution was 0.011. The mean difference of the $D_{\max}$ distribution was 0.11, and 99% of the maximum differences of all 60 × 60‐pixel windows were under 0.22 (99th percentile of $D_{\max}$ distribution). The descriptive statistics of the distribution are displayed in Table 1.

**TABLE 1:** Descriptive statistics for the confidence differences for both $D_{60\times 60}$ and $D_{\max}$ distributions.

	Confidence differences for the $D_{60\times 60}$ distribution	Confidence differences for the $D_{\max}$ distribution
Average	0.011	0.11
Standard deviation	0.021	0.041
25th percentile	0.00042	0.089
50th percentile	0.0029	0.11
75th percentile	0.012	0.14
99th percentile	0.10	0.22
Maximum	0.83	0.83

**FIGURE 3:**
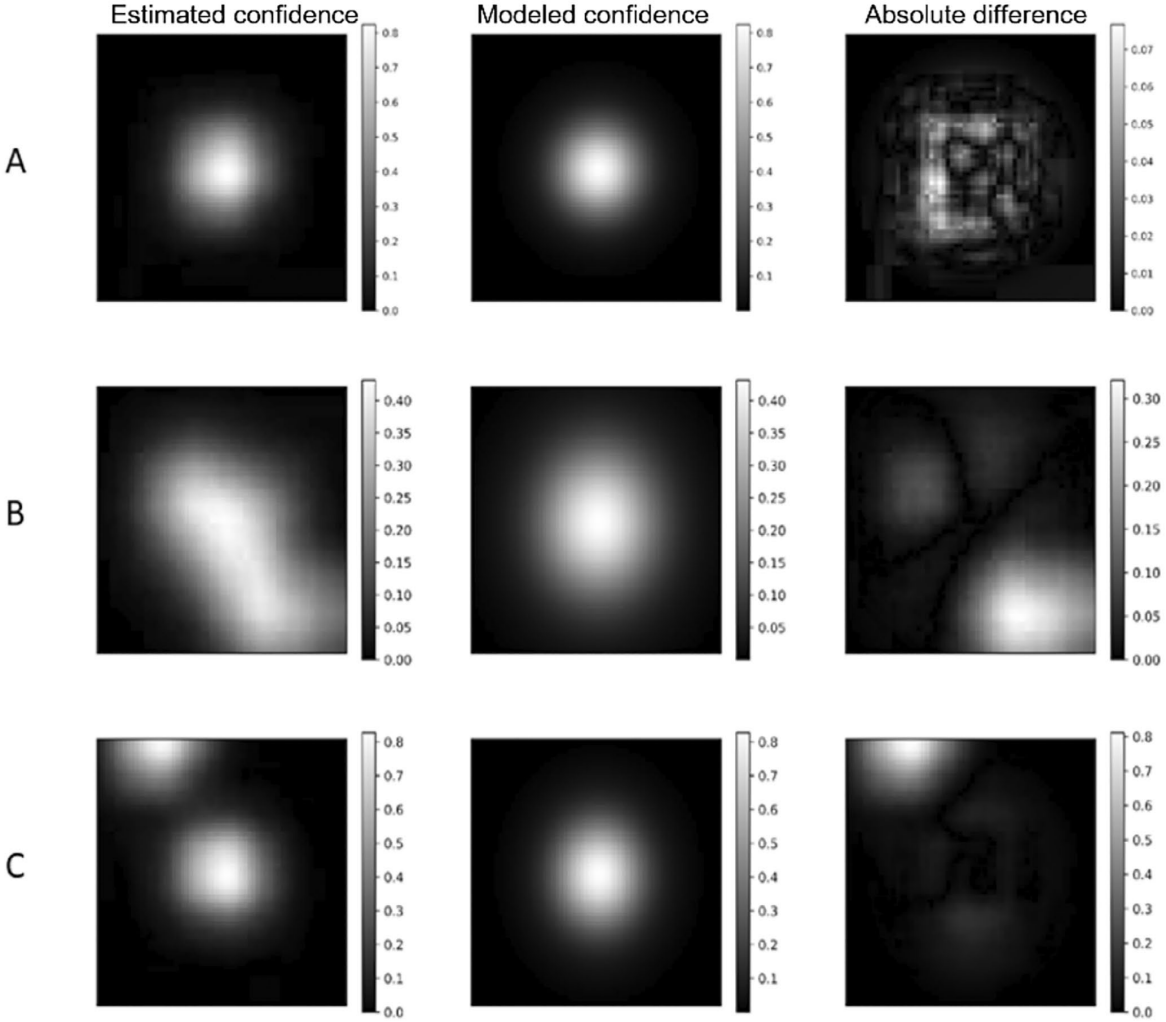
Examples of estimated and modeled 2D confidence heatmaps (respectively left and middle columns), and the associated absolute difference over the 60 × 60 pixel zone (right column). Color scales differ for each plot.

The differences between estimated and modeled confidence heatmaps are presented through three typical cases seen across the data (Figure 3). The first row (Figure 3A) displays the absolute differences for a keypoint which has been estimated with a confidence value bigger than 0.8. The largest differences, which were around 0.07, were located on the side of the confidence zone, while the center of the confidence zone displayed very small differences. This is a typical example of a correctly estimated keypoint, with a high associated confidence value and for which the estimated and modeled 2D confidence heatmaps were very similar.

Larger differences between estimated and modeled heatmaps were found (Figure 3B,C). These cases were very rare: for a total of 361 440 000 pixels analyzed, 66,974 pixels (0.018%) displayed differences above 0.2, and 236 pixels (0.000065%) displayed differences above 0.7. For the case presented in Figure 3B, the differences came from a mismatch between the Gaussian hypothesis for modeling and the estimated confidence. Indeed, the estimated heatmap did not look like a 2D Gaussian, but Gaussian parameters were computed despite this mismatch.

Large confidence differences also came from the presence of multiple estimated high‐confidence areas (Figure 3C). Since only one confidence area is modeled, the other areas contribute to these errors and produce this mismatch.

### 3.2 Evaluation of the Confidence‐Based MKO

Across all cameras, participants, movements, frames, and keypoints, 99.03% of keypoints were estimated by OpenPose. Missing keypoints were mainly caused by self‐occlusions (one body part in front of another) and box‐induced occlusions. Consequently, 10.7% of frames contained at least one missing keypoint, which made it impossible to obtain the 3D points and 3D kinematics for such frames. As a result, joint kinematics could be computed for only 89.3% of frames using the distance‐based MKO. However, joint kinematics could be estimated in 100% of frames using the confidence‐based MKO.

**FIGURE 4:**
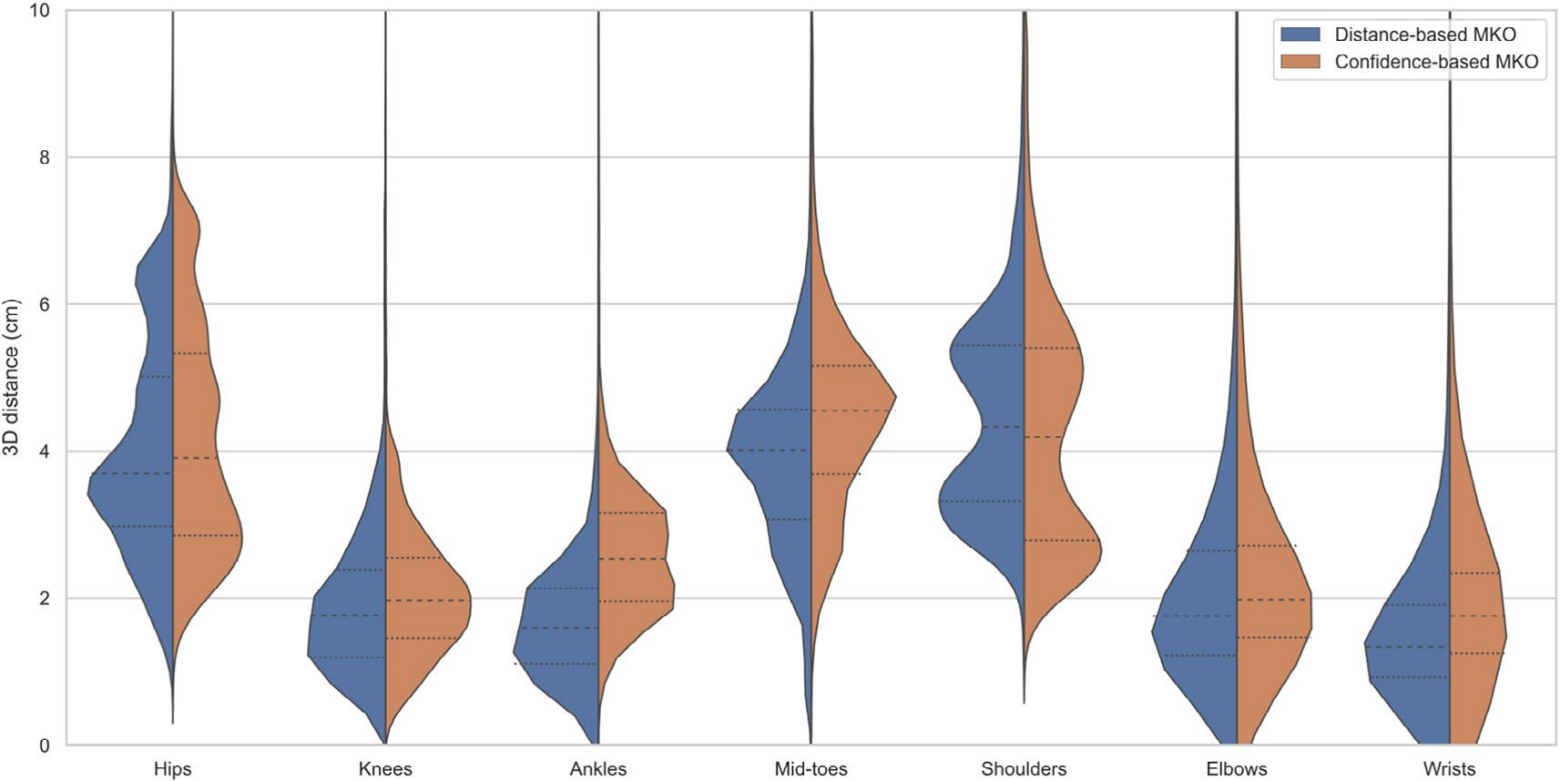
Distribution of 3D Euclidean distances between marker‐based 3D points and markerless 3D points, for both distance‐based MKO (in blue) and confidence‐based MKO (in orange). Only 3D distances smaller than 10 cm have been represented for clarity purposes. Median and quartile values have been represented by dotted lines for each distribution.

The 3D Euclidean distances between reference marker‐based and markerless 3D points were similar for both distance‐based and confidence‐based MKOs. The distributions presented in Figure 4 encompass the 3D Euclidean distances for the 3 performed movements by the 2 participants and all the associated frames. Distributions showed median values between 1.5 and 4.5 cm, depending on the keypoint. The differences in median values between distance‐based and confidence‐based MKOs distributions were of a few millimeters, smaller than 1 cm. The median of the distribution for the distance‐based MKO was smaller than the median of the distribution for the confidence‐based MKO for all 3D points except the shoulders (respectively 4.33 and 4.19 cm).

**FIGURE 5:**
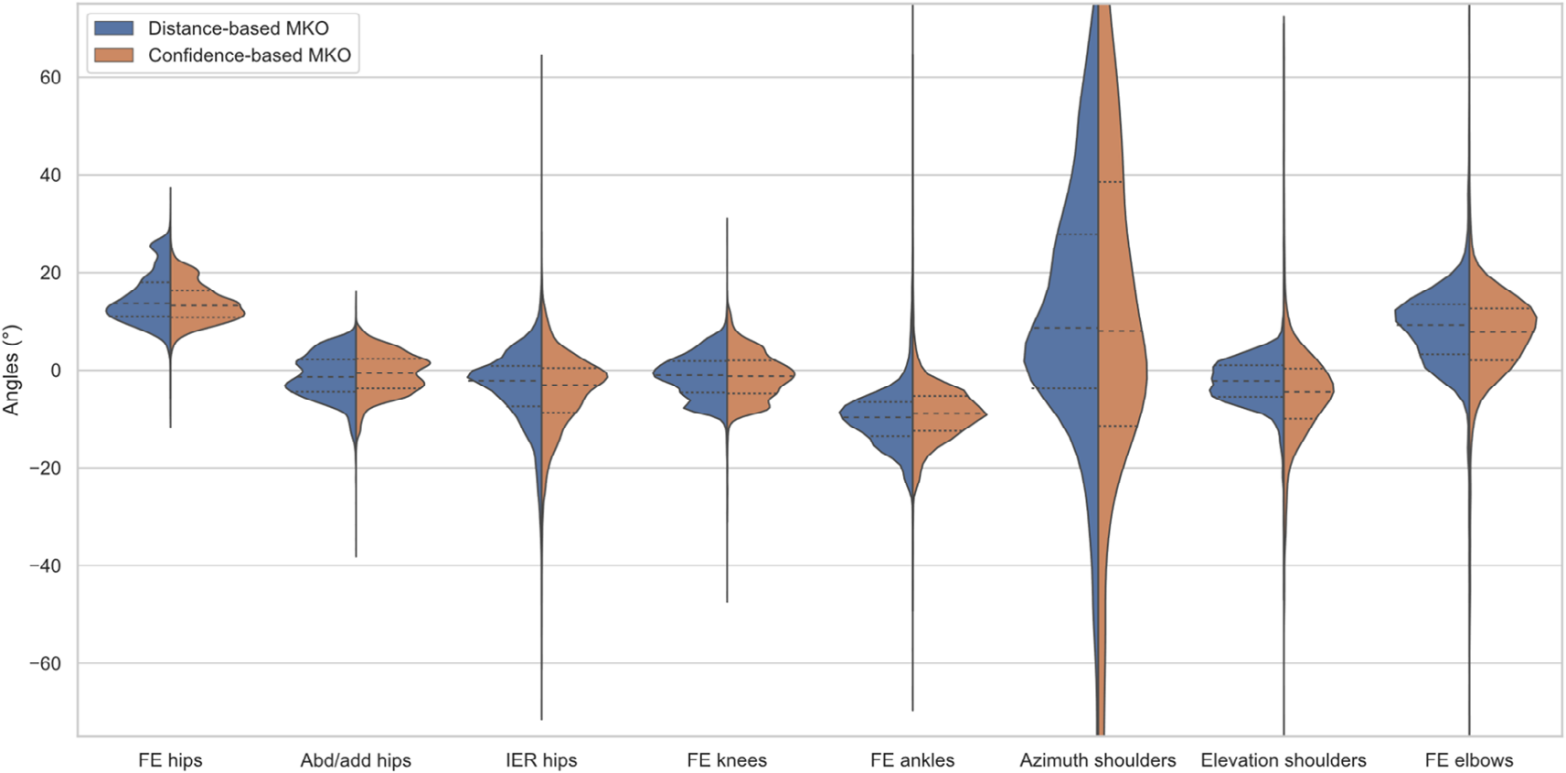
Distribution of angular differences between marker‐based and markerless angles, for both distance‐based MKO (in blue) and confidence‐based MKO (in orange). Only angular differences between −75° and +75° have been represented for clarity purposes. Median and quartile values are represented by dotted lines for each distribution.

The angular differences between reference marker‐based and markerless 3D kinematics were similar for both distance‐based and confidence‐based MKOs. The distributions presented in Figure 5 encompass the angular differences for the 3 performed movements by the 2 participants and all the associated frames. Distributions showed median values between −10° and 13°, depending on the angle. The median of the distribution of the confidence‐based MKO was either smaller (flexion/extension (FE) hips, abduction/adduction hips, FE ankles, azimuth shoulders, FE elbows) or bigger (internal/external rotation (IER) hips, FE knees, elevation of shoulders) than the median of the distribution of the distance‐based MKO angular differences. The differences were less than 1° for all angles except elevation of the shoulders (difference of 2.2°) and FE elbows (difference of 1.4°). Absolute angular differences bigger than 75° were found for both confidence‐based and distance‐based MKO. Using the confidence‐based MKO, these differences accounted for 11.8% (azimuth shoulders), 0.2% (elevation shoulders) and 0.6% (FE elbows) of frames, while for the distance‐based MKO these differences accounted for 2.9% (azimuth shoulders), 0% (elevation shoulders) and 0.09% (FE elbows) of frames.

## 4 Discussion

In this study, we presented the proof of concept of a new confidence‐based MKO method, validated its modeling of 2D confidence heatmaps as Gaussian functions, and evaluated its performance by comparing the resulting 3D kinematics to a reference with a marker‐based approach. Our findings indicate that the 2D Gaussian representation approximates correctly the estimated heatmaps, justifying its use within the confidence‐based MKO. The confidence‐based MKO was able to compute 3D kinematics for every frame, unlike the distance‐based approach, demonstrating its robustness to missing keypoints. Moreover, the 3D kinematics derived from this method were comparable to those obtained via a distance‐based markerless MKO. These results validate the first hypothesis concerning the improved robustness to missing keypoints, while the second hypothesis concerning improved accuracy cannot be validated in light of the obtained results.

### 4.1 Validation of the Gaussian Modeling

The modeling of 2D heatmaps as Gaussian functions draws on established practices in the literature [16, 17], ensuring a fully differentiable objective function. Our results showed a mean absolute difference of 0.011 between the measured and modeled confidence values, which is small relative to the confidence range (0–1). Occasional larger discrepancies arose when the heatmaps were more ellipsoidal than circular or contained multiple high‐confidence regions. These issues could potentially be mitigated by refining the model to include additional parameters, either to capture ellipsoidal shapes and orientation of principal axes or by adopting a top‐down pose estimation framework [16]. Nevertheless, these cases were rare: 99% of the maximum differences within the 60 × 60‐pixel windows remained below 0.22. Consequently, our simplified four‐parameter approach was sufficiently accurate for the confidence‐based MKO, as these small discrepancies would be expected to have a negligible impact on the resulting 3D kinematics, which we discuss in the next section.

### 4.2 Evaluation of the Confidence‐Based MKO

Our first hypothesis was that using the whole confidence information contained in 2D confidence heatmaps could be beneficial to computing joint kinematics for markerless motion capture by improving robustness to missing keypoints. This was validated by the full computational coverage (100% of frames) obtained by the confidence‐based MKO. It outperformed the distance‐based approach, which failed to resolve kinematics in 10.7% of frames due to missing keypoints, even though keypoint trajectories were gap‐filled following standard procedures. Without this gap‐filling procedure, the proportion of frames for which the kinematics could not be obtained would surely have been higher. The robustness of our confidence‐based MKO to missing keypoints stems from processing OpenPose outputs directly in camera planes, rather than in 3D space. Thus, if information is available for only one camera, the confidence‐based MKO can still be run. Additionally, because anthropometry is established beforehand in the rigid biomechanical model, the optimization still maintains consistent constraints even when a 2D keypoint is missing. For the distance‐based MKO, triangulation requires information from at least two cameras: if a keypoint is estimated for only one camera, the triangulation cannot be performed. In the case of a limited number of available 3D points (25 only for OpenPose model *body_25b*), if one 3D point is missing, then the MKO becomes under‐constrained.

Our second hypothesis was that using the whole confidence information contained in 2D confidence heatmaps could be beneficial to computing joint kinematics for markerless motion capture by improving accuracy. Overall, the differences obtained were very close to those obtained with the markerless distance‐based MKO, showing no substantial differences with the introduced method. The similarities of the 3D kinematics obtained with confidence‐based and distance‐based 3D kinematics are surprising, considering the improved MPJPE (mean per joint position error) found by Iskakov et al. [24] when using 3D confidence heatmaps instead of weighted DLT. However, they computed 3D points without implementing an optimization, which involves additional constraints for the final position of the biomechanical model. Thus, the optimization approach might minimize the differences due to the type of data and the associated processing (triangulation versus 2D Gaussian modeling). A previous study of the confidence‐based MKO on synthetic data [25] also showed an improvement in the joint angles using the confidence‐based MKO compared to the distance‐based MKO. However, in this previous study, the 2D keypoints were deliberately estimated at 80 pixels from their expected position. This estimation far from its expected position did not seem to occur frequently in our experimental situation, which could justify the differences between studies.

Angles of the upper arms displayed the largest differences, with differences larger than 75° for some frames, for both confidence‐based and distance‐based MKOs. These differences can be explained by the difficulty to orient properly the arm segments with only three keypoints—shoulders, elbows, wrists. This is especially true for interpretation of the azimuth angle when there was no elevation, which happened when the arm was close to the body. In this situation, a slight difference in the upper arm orientation might completely change the azimuth angle, leading to the large differences observed. Such issues were inexistent for the legs, for which foot keypoints helped define global orientation. Available hand models could be used to try and solve this issue, although this would be a time‐consuming solution. Another possibility would be to implement marker augmentation algorithms, such as the ones used in OpenCap [4, 26], to have additional markers at the upper arms. Some of the differences obtained, whether for 3D distances or angular differences, could be explained by markerless and marker‐based biomechanical model differences. These differences and their impact on the resulting 3D kinematics differences have been observed in the literature, especially for the hip flexion/extension angle [1, 27]. In our study, these differences can be observed for angular differences distributions for the flexion/extension of the hips and elbows, azimuth of the shoulders and flexion/extension of the ankles. These distributions have median values centered on positive or negative angular differences, whereas all the other angular differences are centered on 0°. Having a limited number of points per segment due to the use of OpenPose *body_25b* model forced us to build the associated segments' reference frames differently from what was done using marker data, which led to these constant offsets. The way of building both markerless and marker‐based models was shown by Kanko et al. [27] to influence the 3D kinematics differences, these differences being reduced when the models are built similarly.

The main limitations of this evaluation were the limited number of involved participants and the experimental conditions. The participants both had low BMI, were young and healthy, and thus could not be considered as representative of the general population [28]. The experimental conditions were tailored to suit marker‐based motion capture and were thus also optimal for markerless motion capture. However, our study is a proof of concept, aiming at presenting a new method and preliminary results. Having two participants allowed us to evaluate the feasibility of our method and to show promising results.

Regarding the experimental conditions, they could not entirely be considered as real‐world scenarios because of the use of marker‐based motion capture: the participants wore their underwear, and the movements occurred in the center of a well‐lit experimental room, without moving with respect to the camera. These parameters probably made it easier for the pose estimation algorithm, and it leads us to wonder if the same results would be attainable in other experimental conditions. However, these issues are related to pose estimation methods, and the evaluation of their impact on the 3D kinematics is out of the scope of this paper. Moreover, the markerless distance‐based MKO suffered from the same limitations, thus the comparison between methods could be considered as fair. More thorough evaluation of the impact of confidence and/or gaussian center position errors can be found in [25].

This study is a proof of concept of the proposed markerless confidence‐based method. It proved the technical feasibility of the method using markerless pose estimation and highlighted situations for which this method could be a more robust alternative to distance‐based methods. Although not detailed here, the confidence‐based method could also be beneficial when using top‐down pose estimation methods, which are more sensitive to partial occlusions than bottom‐up methods [29, 30]. It could also be used to better compute 3D kinematics of points that are on the margin of the capture volume, as they might be seen only by a subset of the available cameras. Finally, the first part of the method, consisting of performing the MKO in the 2D plane of the camera instead of working on the 3D point reconstructed by DLT, could advantageously be exploited in any method detecting or estimating points in a 2D camera plane, including the marker‐based approaches. It could thus be used, without the Gaussian modeling, for processing marker‐based data.

## 5 Conclusion

In this study, a novel confidence‐based MKO method was presented for markerless motion capture. The details of this method were provided. A core hypothesis of this MKO method, namely the modeling of the 2D confidence heatmaps as 2D Gaussian functions, was validated, and the confidence‐based MKO method was evaluated by comparing the obtained 3D kinematics to reference values. This evaluation showed that the confidence‐based MKO was more robust to missing keypoints than the distance‐based MKO. However, the hypothesis of improved accuracy when using the confidence‐based MKO could not be verified, as the obtained 3D kinematics were similar to those obtained with the distance‐based MKO.

## Conflicts of Interest

The authors declare no conflicts of interest.

## Data Availability

The data that support the findings of this study are openly available in Benchmarking dataset for markerless motion capture analysis at https://entrepot.recherche.data.gouv.fr/dataset.xhtml?persistentId=doi:10.57745/LQI2MJ, reference number https://doi.org/10.57745/LQI2MJ.
